# Selective Activation of Endoplasmic Reticulum Stress by Reactive-Oxygen-Species-Mediated Ochratoxin A-Induced Apoptosis in Tubular Epithelial Cells

**DOI:** 10.3390/ijms222010951

**Published:** 2021-10-11

**Authors:** Chong-Sun Khoi, Yu-Wen Lin, Jia-Huang Chen, Biing-Hui Liu, Tzu-Yu Lin, Kuan-Yu Hung, Chih-Kang Chiang

**Affiliations:** 1 Graduate Institute of Toxicology, College of Medicine, National Taiwan University, Taipei 10617, Taiwan; d05447005@ntu.edu.tw (C.-S.K.); r04447005@ntu.edu.tw (Y.-W.L.); f04447010@ntu.edu.tw (J.-H.C.); biingliu@ntu.edu.tw (B.-H.L.); 2 Department of Anesthesiology, Far-Eastern Memorial Hospital, New Taipei City 22060, Taiwan; drling1971@gmail.com; 3 Department of Internal Medicine, College of Medicine and Hospital, National Taiwan University, Taipei 10617, Taiwan; kyhung@ntu.edu.tw; 4 Department of Integrated Diagnostics & Therapeutics, National Taiwan University Hospital, Taipei 100225, Taiwan

**Keywords:** ochratoxin A, ER stress, apoptosis, reactive oxygen species, HK-2 cell

## Abstract

Ochratoxin A (OTA), one of the major food-borne mycotoxins, impacts the health of humans and livestock by contaminating food and feed. However, the underlying mechanism of OTA nephrotoxicity remains unknown. This study demonstrated that OTA induced apoptosis through selective endoplasmic reticulum (ER) stress activation in human renal proximal tubular cells (HK-2). OTA increased ER-stress-related JNK and precursor caspase-4 cleavage apoptotic pathways. Further study revealed that OTA increased reactive oxygen species (ROS) levels, and N-acetyl cysteine (NAC) could reduce OTA-induced JNK-related apoptosis and ROS levels in HK-2 cells. Our results demonstrate that OTA induced ER stress-related apoptosis through an ROS-mediated pathway. This study provides new evidence to clarify the mechanism of OTA-induced nephrotoxicity.

## 1. Introduction

Ochratoxin A (OTA) is a mycotoxin which is mainly produced by several species of *Aspergillus* and *Penicillium* [1]. Mycotoxins are secondary metabolites of fungi that can cause disease and death in humans and animals [2]. OTA is widely found in various foods such as corn, wheat, barley, coffee beans, oilseeds, beer, grapes, wine, and animal feed [3,4,5,6]. Because OTA is a common contaminant of food and feed, humans and animals are frequently exposed to OTA in their daily lives [7]. Previous experiments have confirmed that exposure to OTA can lead to multifarious deleterious effects, including hepatotoxicity [8], teratogenicity [9], and nephrotoxicity [10]. A recent study revealed that OTA induced hepatoxicity by increasing oxidative stress, inflammation, and necrosis, and by inhibiting antioxidant enzymes in rat liver tissue [11]. The kidney is the primary target of OTA [12], and the mechanisms that underly OTA nephrotoxicity include the inhibition of protein synthesis, DNA damage, cell cycle arrest, and cell apoptosis [13]. OTA has been recognized as a strong nephrotoxin that accumulates in proximal tubule epithelial cells and initiates cellular damage through oxidative stress, DNA damage, apoptosis, and inflammatory response [14]. In addition, OTA was shown to induce apoptosis in HK-2 cells through activation of MEK/ERK1-2 signaling [7].

In the recent literature, it was found that OTA inhibited tumor necrosis factor receptor-associated protein 1 (TRAP1) by inducing the mitochondria-mediated and ER-stress-excited apoptosis of HK-2 cells [15]. OTA attenuated sphingomyelin and cholesterol to disturb lipid raft formation, which induced the apoptosis of HK-2 cells through the PTEN/PI3K/AKT pathway. Darbuka et al. revealed that OTA promoted the translocation of NFkB into the nucleus, which induced the phosphorylation of ERK1/2 and apoptosis in HK-2 cells [16]. In addition, Longobardi et al. revealed that OTA induced nitrosative stress as well as inflammatory and DNA damage in the kidney and liver of rats [14]. Damiano et al. found that OTA induced oxidative stress, increased glomerular and tubular injury, and tubulointerstitial fibrosis in an in vivo study [17].

The endoplasmic reticulum (ER) is capable of maintaining cellular homeostasis. Newly synthesized and secretory membrane proteins transfer into the endoplasmic reticulum for modification including protein folding, glycosylation, and disulfide bond formation [18]. Numerous environmental, physiological, and pathological insults disturb ER homeostasis, referred to as ER stress [19]. ER stress is evoked in various kidney diseases [20]. Previous studies have shown that OTA could induce ER stress and ROS production, causing glomerular mesangial cell apoptosis [10]. OTA was also shown to induce ER stress in the kidney and spleen of pigs [21].

The roles of ER stress in OTA-induced renal proximal tubular cell cytotoxicity remain unclear. This study investigates the acute injury induced by OTA on human renal proximal tubular cells, and further explores the correlation between nephrotoxicity and ER stress. The present study shows that OTA induces ER stress and ROS in proximal tubular cells, inhibits ROS production, and could reduce the apoptosis of proximal tubular cells during OTA nephrotoxicity.

## 2. Results

### 2.1. OTA Reduced Cell Viability and Induced HK-2 Cells Apoptosis

**Figure 1:**
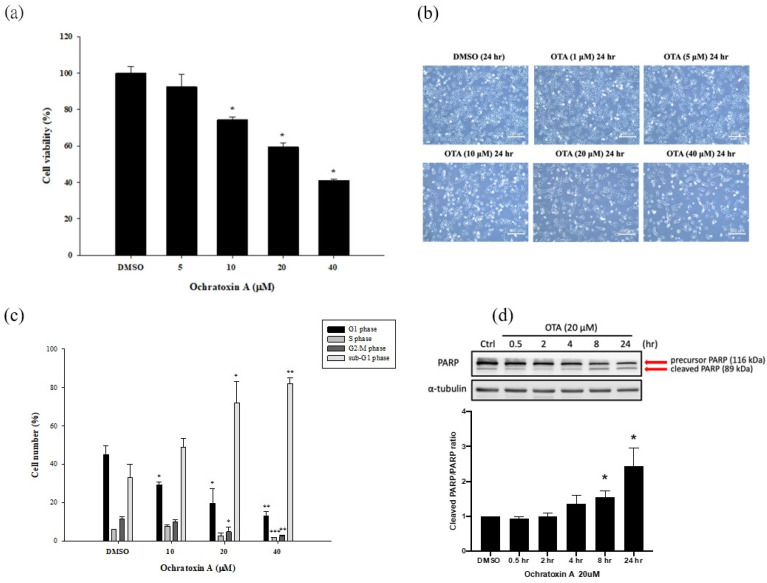
OTA reduced cell viability and induced HK-2 cells apoptosis. (**a**) OTA-treated HK-2 cells viability was detected by MTS assay. (**b**) Morphology and phenotype of HK-2 cells were observed by inverted microscope. (**c**) Cell cycle, and a sub-G1 fraction of OTA-treated HK-2 cells were analyzed by flow cytometry. (**d**) OTA-induced PARP cleavage was time dependent. The statistical analysis was performed using one-way ANOVA followed by Tukey’s multiple comparison test. Data are represented as mean ± S.E.M. N = 3 for each group; * *p* < 0.05, ** *p* < 0.01 and *** *p* < 0.001 as compared to the DMSO group.

Culture media containing DMSO (control) and 1, 5, 10, 20, and 40 μM of OTA were applied to HK-2 cells. After 24 h of culture, the cell survival rate was detected using the MTS test. The cell viability was significantly reduced to 70% compared to DMSO at the lowest concentration of 10 μM, and to 40% at the highest concentration of 40 μM (Figure 1a). Based on this observation, we then observed the morphology of HK-2 cells in different concentrations of OTA for 24 h. In the control group, HK-2 cells maintained their normal growth and morphology. In addition, HK-2 cells showed a series of typical morphological features of apoptosis, such as cell shrinkage and fragmentation into membrane-bound apoptotic bodies, in a dose-dependent manner (Figure 1b). The morphological changes were most apparent in the 20 and 40 μM OTA treatment groups. Because of the excessive apoptosis in the 40 μM OTA treatment group, we selected the concentration of 20 μM OTA for subsequent experiments. To estimate the OTA-induced apoptosis, we applied flow cytometry to analyze PI-stained HK-2 cells. The proportion of cells in the sub-G1 phase increased significantly in a concentration-dependent manner (10–40 μM) after 24 h of exposure (Figure 1c). These findings suggest that OTA-treated HK-2 cells suffered from cellular fragmentation. Western blot analysis proved that the induction of the PARP cleaved form was time dependent (0.5–24 h) after OTA treatment. It was statistically significant after 8 h and 24 h of OTA treatment (Figure 1d). Based on these findings, we conclude that OTA decreased the survival of HK-2 cells by activating the apoptotic pathway.

### 2.2. OTA Induced ER Stress in HK-2 Cells

**Figure 2:**
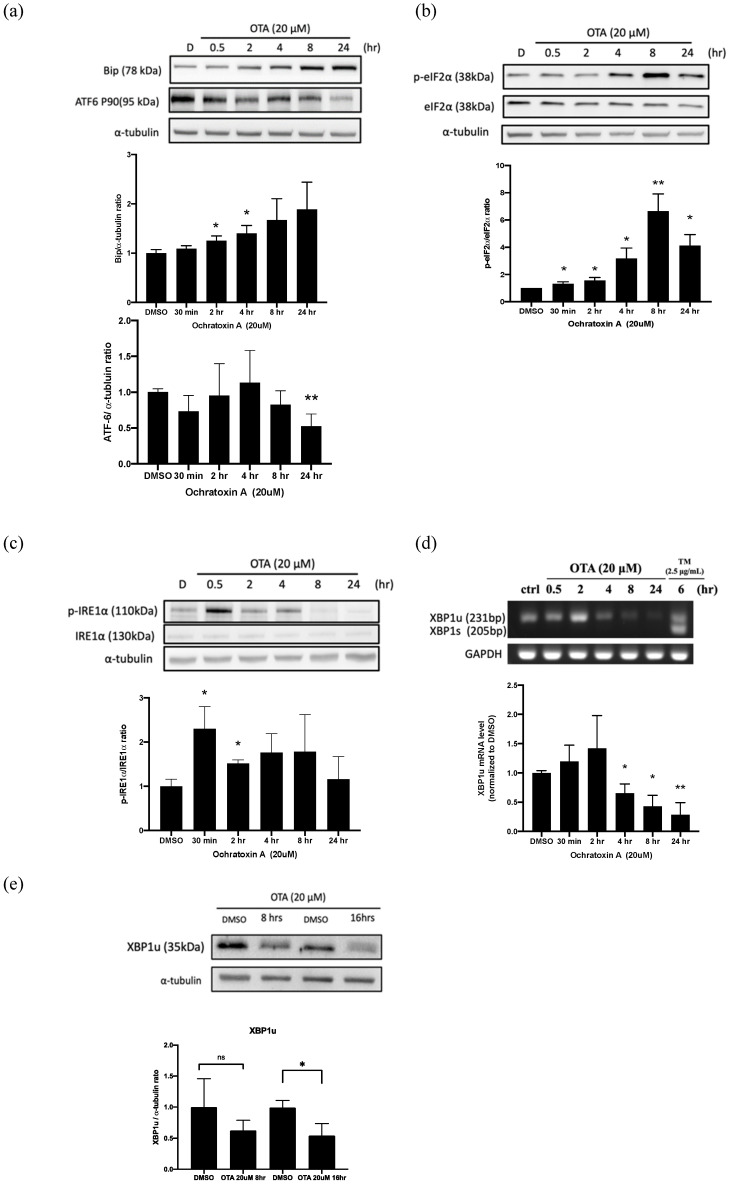
OTA induced ER stress in HK-2 cells. Detection of OTA-induced ER stress in HK-2 cells by Western blot analysis. Protein levels of ER stress surrogate markers, including (**a**) BiP and 90 kDa ATF6; (**b**) p-eIF2α; (**c**) p-IRE1α; and (**e**) XBP1 determined by Western blot analysis. (**d**) XBP1s and XBP1u were expressed by RT-PCR product electrophoresis. The statistical analysis was performed using one-way ANOVA followed by Tukey’s multiple comparison test. Data are represented as mean ± S.E.M. N = 3 for each group; * *p* < 0.05 and ** *p* < 0.01, as compared with control/DMSO group.

Several studies have established that the induction of binding immunoglobulin protein (BiP) is a marker for ER stress. It is a central regulator for ER stress due to its role as a major ER chaperone with anti-apoptotic properties and its ability to control the activation of transmembrane ER stress sensors (ATF6, IRE1α, and PERK) through a binding-release mechanism [22]. We clearly showed that 20 μM OTA enhanced the BiP induction in a time-dependent manner (Figure 2a). ER stress induction led to the activation of the proteolytic cleavage of membrane-bound 90 kDa ATF6 to a 50 kDa protein with transcription-activation properties [23]. As shown in Figure 2a, ATF6 (P90) showed a time-dependent decline, especially at the 24 h time point, with 20 μM of OTA treatment. Moreover, eIF2α phosphorylation led to an inhibition of translation initiation, which reduced the unfolded protein influx into the ER. The results showed that p-eIF2α (Figure 2b) time-dependently increased after OTA treatment. Furthermore, IRE1α phosphorylation (Figure 2c) was robustly induced at the early time point (30 min) with 20 μM OTA treatment, but with no obvious splicing XBP1 induction, as shown by RT-PCR (Figure 2d) and protein blotting (Figure 2e). These findings suggest OTA induced ER stress in a compound-specific manner in HK-2 cells.

### 2.3. OTA Induced ER Stress-Dependent Apoptosis through JNK Activation and Caspase-4 Cleavage

**Figure 3:**
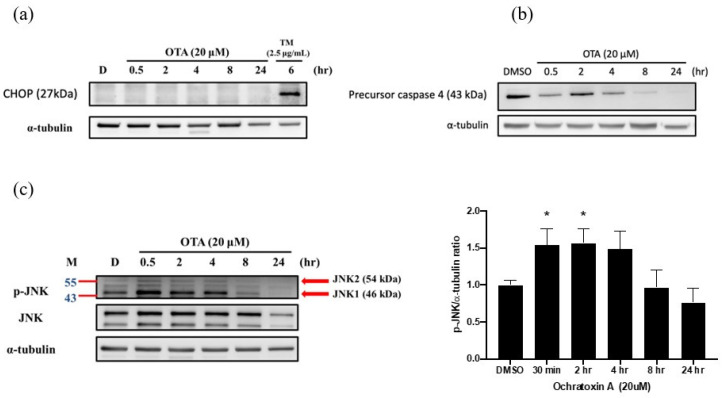
OTA induced ER-stress-dependent apoptosis through JNK activation and caspase-4 cleavage in HK-2 cells. Western blot analysis was performed to detect ER-stress-related apoptotic pathways. Protein expression of (**a**) CHOP, (**b**) precursor caspase-4, and (**c**) JNK after OTA treatment at different time points. The statistical analysis was performed using one-way ANOVA followed by Tukey’s multiple comparison test. Data are represented as mean ± S.E.M. N = 3 for each group; * *p* < 0.05 as compared with DMSO group.

Several pathways contributed to ER-stress-dependent apoptosis during persistent cellular stress [24], such as the C/EBP homologous protein (CHOP), which is also known as growth arrest and DNA damage-inducible gene 153 (GADD153). To explore the potential contribution of CHOP-dependent apoptosis, we treated HK-2 cells with OTA and observed the time-dependent expression for 24 h. Tunicamycin (TM) acted as a positive control of CHOP. There was no CHOP induction in the OTA treatment (Figure 3a). In addition, we further investigated the IRE1 axis of ER stress-mediated apoptosis, including c-JUN NH2-terminal kinase (JNK) and caspase-4 activation [25]. We found that OTA exposure induced precursor caspase-4 cleavage (Figure 3b). Additionally, JNK was robustly phosphorylated after 30 min of OTA treatment, and was dephosphorylated after 8 h, and was continued to be observed throughout (Figure 3c). Furthermore, the XBP1 axis of adaptive UPRs was further explored to confirm the potential activation of the pro-survival pathway. As shown in Figure 2d,e no XBP1s was detected in the OTA exposure in RT-PCR. In addition, the total XBP1u protein levels were also suppressed during OTA treatment. We speculate that OTA induced apoptosis through ER-stress-mediated JNK and caspase-4 activation, but not CHOP-mediated activation. Adaptive XBP1 was selectively inactive in OTA-mediated apoptosis.

### 2.4. ROS Mediated OTA-Induced JNK Activation and Apoptosis in HK-2 Cells

**Figure 4:**
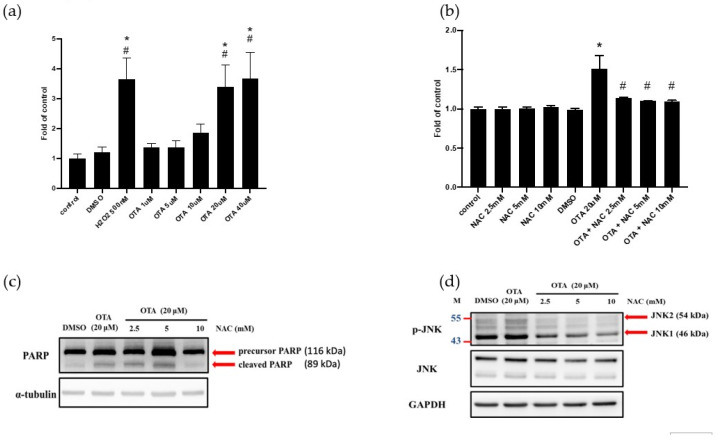
ROS mediated OTA-induced JNK activation and apoptosis in HK-2 cells. DCFDA fluorescence was used to detect intracellular ROS in HK-2 cells. (**a**) Intracellular ROS in HK-2 cells treated with different concentrations of OTA. (**b**) Intracellular ROS in HK-2 combined with NAC and OTA. Protein expression of (**c**) PARP (**d**) JNK after combination treatment with NAC and OTA. The statistical analysis was performed using one-way ANOVA followed by Tukey’s multiple comparison test. Data are represented as mean ± S.E.M. N = 3 for each group; * *p* < 0.05, as compared with control group; # *p* < 0.05, as compared with DMSO.

Previous studies have shown that OTA induces ROS in renal cells [26,27,28]. Elevated ROS activated JNK and led to cell death from apoptosis to necrosis [29]. Therefore, we further investigated whether ROS could activate JNK phosphorylation after OTA treatment. We applied the DCFDA detection assay to analyze the intracellular ROS. The results showed that OTA increased the ROS in HK-2 cells in a concentration-dependent manner (Figure 4a). Whether ROS was involved in OTA-induced apoptosis, N-acetyl cysteine (NAC, an ROS scavenger) was used to elucidate the effect of ROS on cells. We found that different concentrations of NAC inhibited ROS after OTA treatment (Figure 4b). The cleaved form of PARP was decreased in the NAC (10 mM)-treated group of OTA-treated HK-2 cells (Figure 4c). NAC also inhibited the expression of OTA-induced p-JNK (Figure 4d). We speculate that OTA-induced renal tubular epithelium apoptosis can be activated by the ROS-mediated overwhelming of ER stress, positively contributing to HK-2 cell injury.

## 3. Discussion

**Figure 5:**
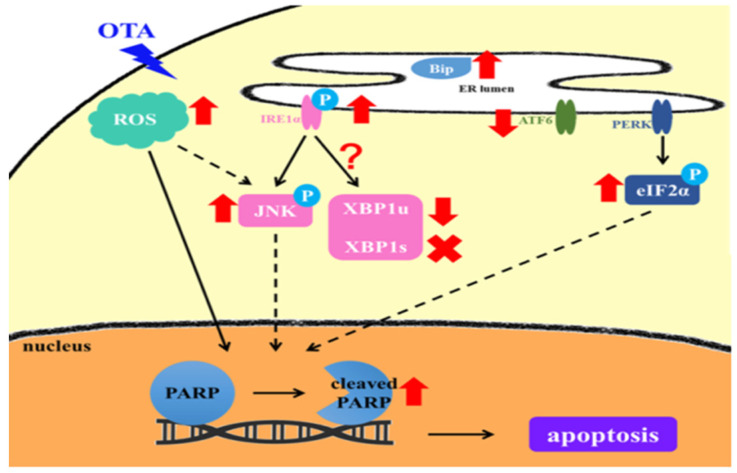
Schematic diagram depicting the mechanisms for OTA-induced tubular cell injury through ROS-mediated ER stress induction. OTA is uptake by HK-2 cells, followed by the robust induction of ROS and consequential selective ER stress activation. The mechanism includes IRE1-mediated JNK activation and caspase-4 cleavage. The figure shows attenuated XBP1 activation and induction, and p-eIF2α-mediated translational shut down without CHOP activation in OTA-treated HK-2 cells.

OTA is a mycotoxin, mainly produced by *Aspergillus ochraceus* and *Penicillium verrucoseum*, and is commonly found in animal feed, corn, and grapes [3,4]. Animals and humans are frequently exposed to it through the consumption of OTA-contaminated food, leading to systemic intoxication or target organ damage [30,31]. To avoid the potential adverse consequences and prevent them properly, we explored OTA-induced renal tubular epithelial injury, especially emphasizing the roles of ER-stress-mediated injuries. We first found that OTA was taken up by HK-2 cells, followed by the robust induction of ROS and consequential selective ER stress activation. OTA-treated HK-2 cells demonstrated IRE1-mediated JNK activation, XBP1 inactivation, and caspase-4 cleavage. OTA treatment also induced p-eIF2α-mediated translational shut-down without CHOP activation (Figure 5). One of our recent works systemically reviewed the therapeutic approaches targeting proteostasis in kidney disease and fibrosis [32]. Therefore, several points deserve discussion with the aim of finding potential interventions in ER-mediated kidney injury under OTA exposure.

Zhang B et al. showed an OTA-induced cell cycle G0/G1 phase arrest in human proximal tubular cells via DNA methylation alteration [33]. Current evidence suggests that cell cycle arrest is a hallmark of senescence induced by ROS production in renal tubular cells. In our study, we further found that OTA enhanced apoptosis, as shown by increases in the sub-G1 fraction and cleaved PARP after OTA exposure in HK-2 cells (Figure 1c,d). The accumulated evidence suggests that ER stress is evoked in various kidney diseases and contributes to the pathological consequences [20]. Gan F et al. demonstrated OTA-induced ER stress in pigs’ kidneys by increasing the BiP level [21]. In addition, our previous study showed that OTA could induce ER-stress-mediated apoptosis via calpain activation in mesangial cells [10]. Shibusawa et al. showed that ER stress mediated the apoptosis of HK-2 cells through the eIF2α/ATF4/CHOP pathway [34]. In contrast to the contribution of ER stress in kidney injury, Inagi R et al. demonstrated that preconditioning with ER stress attenuated experimental glomerulonephritis [35]. Cybulsky AV et al. stated that ER stress protein induction is a novel protection mechanism from complement attack in passive Heymann nephritis [36]. In this study, we found that OTA selectively activated IRE1α-mediated JNK phosphorylation, which was followed by PARP cleavage and apoptosis in proximal tubular cells (Figure 2c and Figure 3c). The phenomenon could be attenuated by NAC, suggesting that OTA induced ER-stress-mediated apoptosis through ROS-dependent pathways.

The p-eIF2α in the PERK pathway increased significantly after OTA treatment (Figure 2b), which showed that the PERK pathway was indeed activated. However, OTA did not lead to the p-eIF2α downstream induction of CHOP (Figure 3a). We speculate that the activation of p-eIF2α did not move toward the path of inducing ATF4 expression, but tended to inhibit overall translation. Similarly, ATF6 (P90) significantly declined until 24 h after OTA exposure. The activation of the ATF6 axis at this time point did not support that it participated in the early phase of OTA-induced HK-2 apoptosis (Figure 1c and Figure 2a). During the activation of ER stress, IRE1α-mediated pathways play critical roles in cell fate for different cell types. In the early phase of ER stress, the activation of IRE1α enables unconventional splicing of the Xbp1 mRNA by the RNase domain of IRE1α, resulting in a 26-nucleotide frameshift and generating an active transcription factor (XBP1s) [37]. This promotes the adaptive response to ER stress. However, the cytoplasmic part of the IRE1 binds the TNF receptor associated factor 2 (TRAF2), an adaptor protein that couples plasma membrane receptors to JNK activation. This consequently activates the IRE1–JNK-mediated ER-stress-dependent apoptosis [38]. OTA was shown to induce apoptosis in MARC-145, Vero monkey kidney epithelial cells, and HEK293 human kidney epithelial cells via JNK pathways [39]. In addition, OTA could activate JNK and apoptosis in MDCK-C7 cells [40]. Similarly, we demonstrated that JNK was immediately phosphorylated after OTA exposure (Figure 3c). Our findings provide evidence that OTA induced apoptosis, at least partially, through ER-stress-dependent IRE1–JNK-mediated apoptosis.

Schaaf GJ et al. have elegantly shown that OTA induced endogenous ROS induction and GSH depletion in rat and pig kidney epithelial cells [26]. High-concentration OTA exposure was shown to increase ROS levels and oxidative DNA damage in human proximal tubular cell lines [27] and Vero cells [28]. Furthermore, excess ROS induced ER stress after exposure to patulin, a common mycotoxin in fruit products [41]. Liu et al. found that excess oxidative stress induced ER-stress-related apoptosis in the kidneys of aged mice and attenuated the pro-survival IRE1-XBP1 signals [42]. In other words, overwhelming ER stress could induce an ROS cascade through NADPH oxidase 4 and calcium-dependent induction [43]. Our results found that OTA robustly increased ROS production and inhibited IRE1-XBP1 activation (Figure 2d,e and Figure 4a,b). We also found that NAC, a ROS scavenger, inhibited OTA-induced apoptosis of proximal tubular cells (Figure 4c,d). Our previous work demonstrated that 4-PBA, an ER chemical chaperone, successfully ameliorated ER-stress-induced renal tubular cell apoptosis and renal fibrosis [44]. We are interested in applying 4-PBA to clarify the relationship between ER stress and ROS after OTA exposure.

## 4. Materials and Methods

### 4.1. Cell Culture

Human renal proximal tubular epithelial cells (HK-2) were obtained from American Type Culture Collection (Manassas, VA, USA). The HK-2 cells experiment was approved by the Biosafe community of National Taiwan University (number: BG1060084). HK-2 cells were cultured in DMEM/F12 (Gibco, CA, USA) medium supplemented with 10% FBS in a 37 °C constant-temperature incubator containing 5% carbon dioxide.

### 4.2. OTA Treatment

Ochratoxin A (OTA) was purchased from SIGMA-ALDRICH (O1877). OTA was dissolved in DMSO with a final concentration of 20 mM and stocking. OTA was diluted to 1/1000 with culture medium during the experiment, and the final concentration of DMSO was 0.1% *v*/*v* in medium. DMSO at a concentration of 0.1% was used as the control for the subsequent experiments.

### 4.3. MTS Assay

The MTS assay determined cell viability. The 3-(4,5-dimethylthiazol-2-yl)-5-(3-carboxy-methoxyphenyl)-2-(4-sulfophenyl)-2H-tetrazolium (Promega, Wisconsin, USA) was reduced by NAD(P)H-dependent dehydrogenase enzymes to generate blue-violet crystals (formazan); 1 × 10^4^ HK-2 cells were cultured in a 96-well culture plate. After 16 h, fresh medium containing 0, 10, 25, and 50 μM OTA was added. After 24 h, MTS was added for 40 min of culture, and finally, the amount of formazan was measured by the absorbance at a wavelength of 490 nm by a Paradigm Multi-Mode Plate Reader (Beckman Coulter PARADIGM).

### 4.4. Intracellular Reactive Oxygen Species (ROS) Assay

2′,7′-Dichlorodihydrofluorescein diacetate (DCFDA) is a fluorescent dye used to detect reactive oxygen species (ROS) content. HK-2 cells (1 × 10^4^) were cultured in a 96-well culture dish. After 16 h, DMEM (without phenol red) medium containing 10 μM DCFDA was added, and the sample was returned to the 37 °C incubator. After 1 h, the medium containing DCFDA was removed and washed twice with PBS. Then, medium containing 0, 1, 5, 10, 20, and 40 μM OTA was added. After culturing for 24 h, a continuous multi-function microplate was used in Paradigm Multi-Mode Plate Reader to detect fluorescence intensity at an excitation wavelength of 485 nm and 535 nm.

### 4.5. Cell Cycle Analysis by Flow Cytometry

Following this, 1.8 × 10^5^ HK-2 cells were exposed to OTA for 24 h, followed by centrifugation and removal of the supernatant, and then the addition of PBS, RNase, and propidium iodide. Meanwhile, methanol was added to fix DNA. This reaction was protected from light at room temperature for 30 min and finally analyzed by flow cytometry (FACS LSR II, Becton Dickinson).

### 4.6. Semi-Quantitative Polymerase Chain Reaction (Semi-Quantitative PCR)

cDNA was obtained with reverse transcription. Subsequently, Taq Master Mix (Bio-Genesis) and primers were added. The mixture was added to a polymerase chain reaction reactor (Applied Biosystems TM 2720 Thermal Cycler) for semi-quantitative PCR. After the reaction, agarose gel electrophoresis was performed, followed by a UV-ray digital image analysis. The following primers were used: XBP1 primer (forward): 5′-gTCTgCTgAgTCCgCA-3′, (reverse): 5′-TCCTTCTgggTAgACCTCTgggAg-3′; and GAPDH primer (forward): 5′-ggTggTCTCCTCTgACTTCAACA-3′, (reverse): 5′-TTCACCACCATggAgAAggC-3′.

### 4.7. Western Blotting Assay

HK-2 whole-cell lysate was performed using radioimmunoprecipitation assay (RIPA) lysis buffer (Cell Signaling Technology) for total cell protein extraction. We collected the original culture solution to centrifuge, which was followed by removing the supernatant, and was then washing with PBS. The Coomassie Brilliant Blue G-250 protein quantification method (Bradford protein assay, Bio-Rad) was used to quantify protein concentration. Proteins in cell lysates were separated by sodium dodecyl sulfate-polyacrylamide gel electrophoresis (SDS-PAGE) and transferred to methanol-activated 0.45 μm polyvinyl difluoride (PVDF) membrane (Merk Millipore, MA, USA). The membrane was blocked with 5% milk, then the blots were incubated with anti-phospho-IRE1α (Novus Biologicals, 1:1000), anti-IRE1α (Cell Signaling Technology, 1:1000), anti-phospho-eIF2α (Cell Signaling Technology, 1:1000), anti-eIF2α (Cell Signaling Technology, 1:1000), anti-phospho-JNK (Cell Signaling Technology, 1:2000), anti-JNK2 (Cell Signaling Technology, 1:1000), anti-PARP (Cell Signaling Technology, 1:1000), anti-BiP (Cell Signaling Technology, 1:1000), anti-caspase 4 (GeneTex, 1:1000), and anti-GAPDH (GeneTex, 1:1000). Then the membrane was incubated with HRP-conjugated secondary antibodies. Finally, the membrane was visualized by UVP BioSpectrum 810 (DBA Analytik, Jena, LA, USA) and quantified using ImageJ (NIH, Bethesda, MD, USA).

### 4.8. Statistical Analysis

The experimental results are expressed as the standard error of the mean (mean ± SD). The Student’s *t*-test was used to analyze the two groups. One-way ANOVA and Tukey’s multiple comparison tests were used for the comparison of more than two groups. A *p*-value less than 0.05 was considered statistically significant.

## 5. Conclusions

This study demonstrated for the first time that OTA could induce ER stress, oxidative stress, and apoptosis in proximal tubular cells. Excessive ER stress and oxidative stress could lead to cell death, ultimately via their influence on each other. Further research should modulate ER-stress-related maladaptation to uncover the unmet needs of kidney protection. The inhibition of ER stress and ROS production can reduce cell apoptosis, which could provide a strategy against OTA-induced nephrotoxicity.

## Data Availability

Not applicable.

## Abbreviations

4-PBA: 4-phenylbutyric acid; ATF4: activating transcription factor 4; ATF6: activating transcription factor 6; BiP: binding immunoglobulin protein; CHOP: CAAT/enhancer-binding protein (C/EBP) homologous protein; eIF2α: eukaryotic translation initiation factor 2α; ER: endoplasmic reticulum; ERK1/2: extracellular signal-regulated kinases 1/2; GSH: glutathione; IRE1α: inositol-requiring enzyme 1 α; JNK: c-Jun amino-terminal kinase; MEK: mitogen-activated protein kinase kinase; NAC: N-acetyl cysteine; OTA: ochratoxin A; PARP: poly(ADP-ribose) polymerase; PERK: protein kinase R (PKR)-like endoplasmic reticulum kinase; RNase: ribonuclease; ROS: reactive oxygen species; TRAF2: tumor necrosis factor receptor-associated factor 2; XBP1: X-box binding protein 1; XBP1u: unspliced isoform of XBP1; XBP1s: spliced isoform of XBP1.
